# Supplementary material for: Infection and Persistence of Coxiella burnetii Clinical Isolate in the Placental Environment
Source: Int J Mol Sci. 2023 Jan 7;24(2):1209. doi: 10.3390/ijms24021209 (PMC9866107; doi:10.3390/ijms24021209)
Supplement: Supplementary file 1 [file ijms-24-01209-s001.zip › ijms-2092057-supplementary.pdf]

International Journal of
Molecular Sciences

MDPI

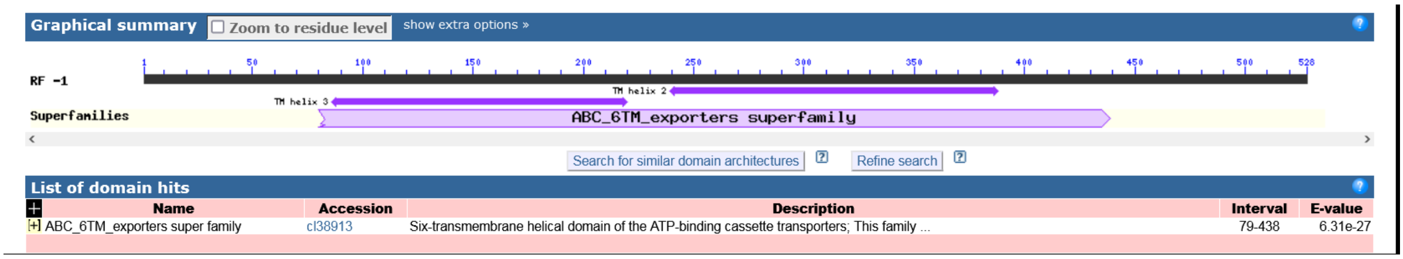

Supplementary Figure S1. Conserved domain searches of protein model Cb122_00594 according to Conserved Domain Database (CDD)[23].

**Protein model**	**Cb122_00594**
**Confidence**	99.94%
**Aligned Residues**	174
**% Identity**	22%
**Template**	c7sgrE_
**PDB info**	**PDB header**:membrane protein<br>**Chain:** E: PDB Molecule:alpha-hemolysin translocation atp-binding protein hlyb<br>**PDBTitle:** structure of hemolysin a secretion system hlyb/d complex
**Resolution**	2.90 Å

>CB122_00594 LVQFFKKFRFQLIQLLIISLLSAALSLAIPLYILIVYDRVVRALSSSMLFSFGGGVLIALIGIALLEKLRSNLMTYIGNQLDASTGREILDKVLSLPMTY LEQSPVGSQIARIKDYDSLRSVITGNLLTLLFDIPVAILFLFVVAFMGGNANLSSYLYRCSFYNIHTVILSAHQ

>c7sgrE_ FIPAIIKYRKIFIETLVVSVFLQLFALITPLFFQVVMDKVLVHRGFSTLNVITVALSVVVVFEIILSGLRTYIFAHSTSRIDVELGAKLFRHLLALPISY FESRRVGDTVARVRELDQIRNFLTGQALTSVLDLLFSFIFFAVMWYYSPKLTLVILFSLPCYAAWSVFISPILR

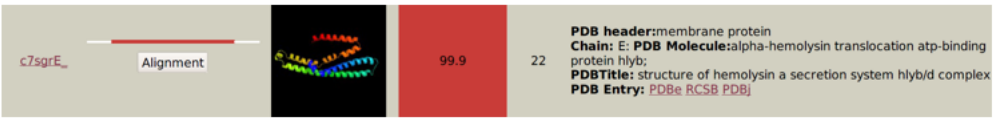

Supplementary Figure S2. Protein modelling of Cb122_00594 according to protein structure prediction Phyre2 [24]. In total, 174 residues have been modelled with 99.9% confidence by the single highest scoring template.

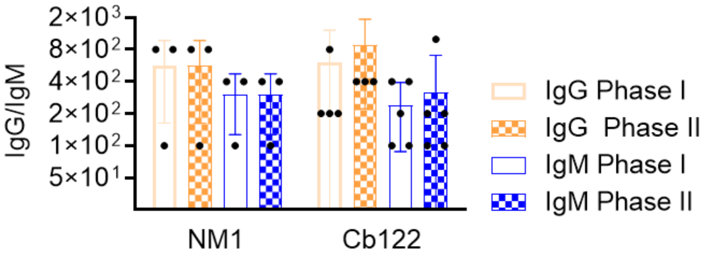

Supplementary Figure S3. Balb/c mice (five mice per group) were infected with *C. burnetii* Cb122 isolate and the NM strain. Eleven days post-infection (A) the humoral response (IgG/IgM phase I/II).

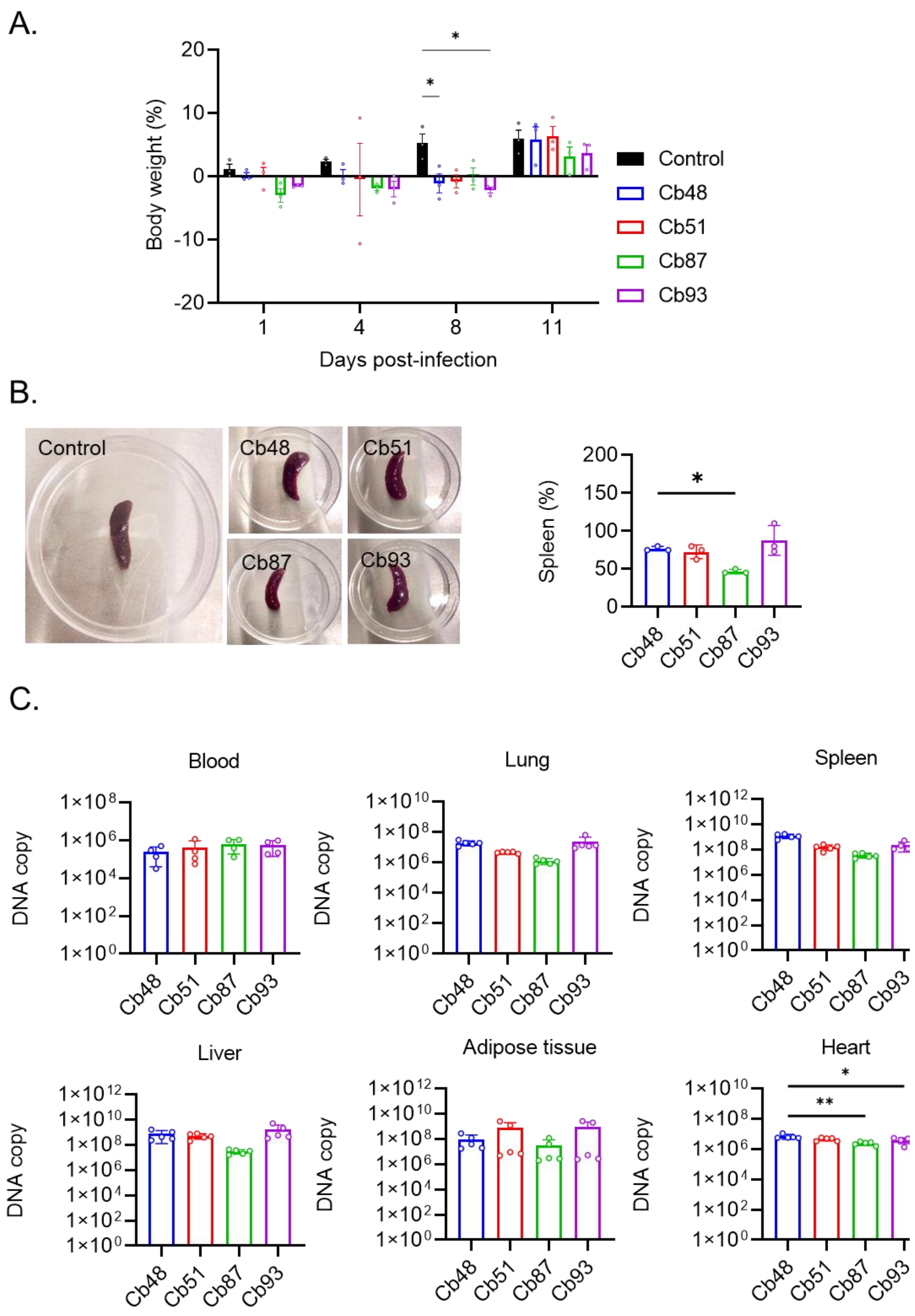

Supplementary Figure S4. Virulence of placental isolates in a murine model of infection. Female Balb/c mice were infected with four *C. burnetii* isolates, Cb48, Cb51, Cb87 and Cb93 (five mice per group). The (**A**) whole body weight of infected mice was monitored every three days. Eleven days post-infection, (**B**) splenomegaly was evaluated using the spleen images (left panel) and percentage of change relative to the control group (right panel). The graph shows a logarithmic scale. The bac-terial burden was evaluated as (**C**) number of DNA copies from 30 mg of lung, liver, adipose tissue, spleen, heart and blood.

Supplementary Table S1. Number of genes from investigated *C. burnetii* isolates. Number of total genes for each investigated *C. burnetii* isolate and reference strain including Cb175 (Guyana), NL3262 (Netherlands), RSA493 (Nine Mile) and Z3055 (German).

	**Total genes**	**Common genes**								
		**Cb48**	**Cb51**	**Cb87**	**Cb93**	**Cb122**	**Cb175**	**NL3262**	**RSA493**	**Z3055**
**Cb48**	2012		1694	1684	1620	1844	1730	1748	1799	1792
**Cb51**	1960			1892	1743	1686	1653	1631	1668	1646
**Cb87**	1950				1739	1679	1647	1631	1647	1633
**Cb93**	1975					1619	1629	1573	1587	1572
**Cb122**	1997						1714	1738	1762	1772
	**Total genes**	**Unique genes**								
		**Cb48**	**Cb51**	**Cb87**	**Cb93**	**Cb122**	**Cb175**	**NL3262**	**RSA493**	**Z3055**
**Cb48**	2012	38	0	0	2	18	8	4	3	2
**Cb51**	1960		21	24	2	1	2	0	1	1
**Cb87**	1950			20	4	2	2	1	0	0
**Cb93**	1975				93	1	37	4	1	0
**Cb122**	1997					44	4	5	1	2

Supplementary Table S2. Analysis of isolates' common genes. Representation number and percentage of isolates genes: present genes, core genes, missing genes, no core genes (isolates' genes shared with reference strains), isolates' unique genes, unique genes for isolates shared with all genes for Cb175 (Guyana), NL3262 (Netherlands), RSA493 (Nine Mile) and Z3055 (German), unique genes for isolates shared with unique genes for Cb175 (Guyana), NL3262 (Netherlands), RSA493 (Nine Mile) and Z3055 (German). The percentage for isolates' present, core and missing genes were related to whole genomes. The percentage for isolates' unique genes were related to present genes in all isolates. The percentage for isolates' unique genes shared with reference strains were related to isolates with no core genes in all isolates.

				**Isolates**		**Unique genes**							
						**Sharing with**				**Unique to**			
	**Present**	**Core**	**Missing**	**No core**	**Unique**	**Cb175**	**NL3262**	**RSA493**	**Z3055**	**Cb175**	**NL3262**	**RSA493**	**Z3055**
**Number**	1521.000	1379;000	870.000	142.000	32.000	48.000	64.000	90.000	77.000	5;000	3.000	4.000	1.000
%	44,202	40.076	25.283	4.127	2.104	33.803	45.070	63.380	54.225	3.521	2.113	2.817	0.704
